# Sensitivity analysis for interpretation of machine learning based segmentation models in cardiac MRI

**DOI:** 10.1186/s12880-021-00551-1

**Published:** 2021-02-15

**Authors:** Markus J. Ankenbrand, Liliia Shainberg, Michael Hock, David Lohr, Laura M. Schreiber

**Affiliations:** Chair of Cellular and Molecular Imaging, Comprehensive Heart Failure Center (CHFC), University Hospital Würzburg, Am Schwarzenberg 15, 97078 Würzburg, Germany

**Keywords:** Deep learning, Neural networks, Cardiac magnetic resonance, Sensitivity analysis, Transformations, Augmentation, Segmentation

## Abstract

**Background:**

Image segmentation is a common task in medical imaging e.g., for volumetry analysis in cardiac MRI. Artificial neural networks are used to automate this task with performance similar to manual operators. However, this performance is only achieved in the narrow tasks networks are trained on. Performance drops dramatically when data characteristics differ from the training set properties. Moreover, neural networks are commonly considered black boxes, because it is hard to understand how they make decisions and why they fail. Therefore, it is also hard to predict whether they will generalize and work well with new data. Here we present a generic method for segmentation model interpretation. Sensitivity analysis is an approach where model input is modified in a controlled manner and the effect of these modifications on the model output is evaluated. This method yields insights into the sensitivity of the model to these alterations and therefore to the importance of certain features on segmentation performance.

**Results:**

We present an open-source Python library (misas), that facilitates the use of sensitivity analysis with arbitrary data and models. We show that this method is a suitable approach to answer practical questions regarding use and functionality of segmentation models. We demonstrate this in two case studies on cardiac magnetic resonance imaging. The first case study explores the suitability of a published network for use on a public dataset the network has not been trained on. The second case study demonstrates how sensitivity analysis can be used to evaluate the robustness of a newly trained model.

**Conclusions:**

Sensitivity analysis is a useful tool for deep learning developers as well as users such as clinicians. It extends their toolbox, enabling and improving interpretability of segmentation models. Enhancing our understanding of neural networks through sensitivity analysis also assists in decision making. Although demonstrated only on cardiac magnetic resonance images this approach and software are much more broadly applicable.

## Background

Image segmentation is of great interest in medical imaging, e.g. in imaging of tumors [1, 2], retina [3], lung [4], and the heart [5]. In the latter, segmentation is applied to partition acquired images into functionally meaningful regions. Quantitative static and dynamic measures of diagnostic relevance are derived from that. These measures include myocardial mass, ventricular volumes, wall thickness, wall motion and ejection fraction. State-of-the-art performance for automatic segmentation is achieved with artificial neural networks [6–8].

Additionally, segmentation of pathological tissue is important for quantification and severity assessment. For this purpose, deep learning-based segmentation models of scar tissue after myocardial infarction have been proposed [9].

Many researchers demonstrated impressive performance on their test task and target data. However, neural networks also have limitations, mainly regarding generalization to new data and interpretability [10].

The limited generalization is particularly problematic as both training data and real-world data are rarely from the exact same distribution. Methods to deal with so-called dataset shift are subject of ongoing research [11]. Furthermore, there might be the effect of hidden stratification [12], there is usually some kind of bias in sampling the training data [13] and networks might learn shortcuts [14] using unintended features to boost performance on the training set. This is commonly addressed by using diverse data sources and extensive data augmentation or sophisticated models [15]. Recently, models with inbuilt prediction of segmentation accuracy have been developed in an effort to make AI in medical imaging more transparent and move away from black box models [16, 17]. A general framework to evaluate, quantify and boost generalization is missing.

Explainability and interpretability of neural networks are additional active fields of research [10, 18]. In model interpretability the goal is to understand how and why a model makes certain predictions. While local interpretability describes a certain prediction by the model based on a defined input, global interpretability delineates the understanding of general features determining the models’ predictions. Specifically, for neural networks a variety of methods have been recently developed to determine so-called attribution [19]. Here attribution means evaluating the contribution of input features [20], layers [21] or single neurons [22] to the prediction.

Sensitivity analysis was first proposed by Widrow et al. in the context of misclassification caused by weight perturbations because of noisy input and machine imprecision [23]. Ever since the term sensitivity analysis has been overloaded with different meanings related to each other. Extensive work has been published on the topic of neural network sensitivity to parameter noise [24]. Here we define sensitivity analysis as exploration of the effect of input transformations on model predictions. The most closely related approach to the one presented here uses algorithm sensitivity analysis for tissue image segmentation [25]. This work shares the general idea, however, differs in a variety of factors such as automatic parameter search and its focus on computational performance [25].

In this work, we describe a straightforward method to interpret arbitrary segmentation models. This sensitivity analysis provides intuitive local interpretations by transforming an input image in a defined manner and inspecting the impact of that transformation on the model performance.

It can be used to answer common questions in machine learning projects: can a network, trained and published by someone else, be applied to my own data? Is it necessary or beneficial to prepare the data in a certain way? We demonstrate how these questions can be addressed by sensitivity analysis in the first case study. Other common questions are: how robust is a model that was trained on a limited dataset regarding characteristics of the data (e.g., orientation, brightness)? How problematic are potential perturbations such as image artifacts? An approach to solve these issues is described in the second case study.

In addition to describing the method and highlighting its utility in two case studies, we present an open-source python library called misas (model interpretation through sensitivity analysis for segmentation) that makes it easy to apply sensitivity analysis to new data and segmentation models.

### Implementation

Sensitivity analysis of segmentation models can happen qualitatively and quantitatively. In the qualitative case the segmentation is done on the original input and transformed (e.g., rotated) versions of it. The resulting segmentation masks are presented to the user as overlays on the transformed images for evaluation. In misas there is an option to get the results presented as a static image sequence or as an animated gif. Quantitative sensitivity analysis requires the availability of a ground truth segmentation of the original image. This way the segmentation performance of the model can be judged by calculating a similarity metric between the prediction and truth. Depending on the transformation the ground truth mask remains the same (e.g., brightness and contrast transformations) or needs to be transformed as well (e.g., rotation, zooming, cropping). The calculated score depending on the parameter of the transformation can be plotted. In misas the Dice score for each individual class can be calculated across the parameter range.

The software library described in this article is written in Python 3. The development was achieved by literate programming [26] in Jupyter notebooks using the nbdev framework, which provides all library code, documentation, and tests in one place. The source code is hosted on GitHub (https://github.com/chfc-cmi/misas) and archived at zenodo (https://doi.org/10.5281/zenodo.4106472). Documentation (https://chfc-cmi.github.io/misas) consists of both a description of the application programming interface (API) usage and tutorials, which include the two case studies. Continuous integration is provided by GitHub actions, where any version pushed to the master branch is tested by running all cells of each notebook in a defined minimal environment. Installable packages are released to the python package index (https://pypi.org/) for easy installation. misas integrates multiple open-source projects such as fastai [27], pytorch [28], torchio [29], and numpy [30].

**Fig. 1:**
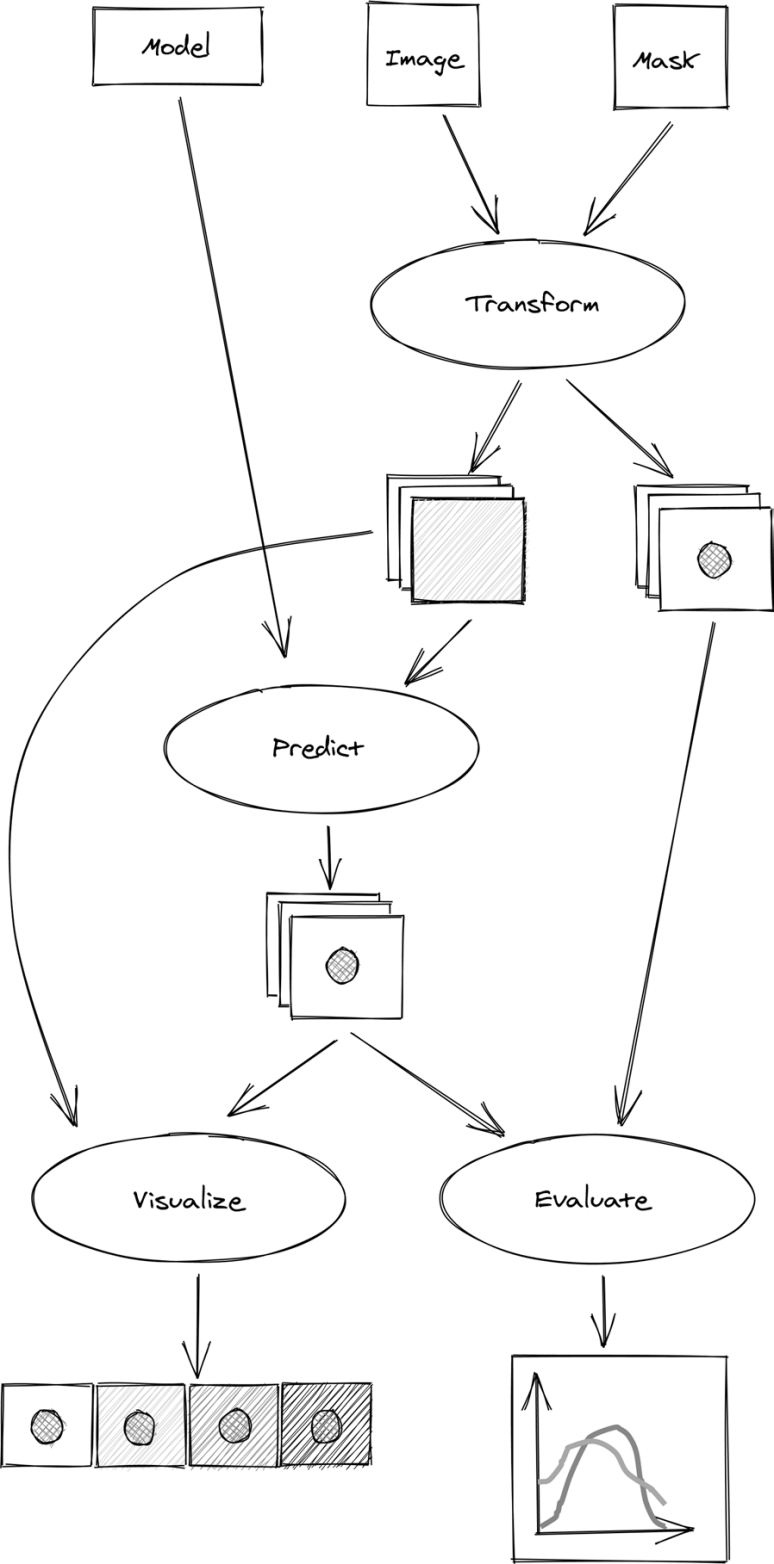
Schematic workflow of misas. Input are a model, an image and optionally a ground truth mask. Images are transformed (e.g. rotated, cropped, zoomed) across a parameter space. Predictions are made on these transformed images and the result is visualized or evaluated using the masks (accordingly transformed if necessary)

The software is generic and framework-independent and was tested with pytorch, fastai v1, fastai v2, and tensorflow [31]. In order to apply misas to new data, images and masks can be imported into misas from a variety of sources, e.g., from png images. The model needs to provide a prediction function that takes an image and returns a predicted segmentation mask (Fig. 1). This can be achieved for an arbitrary model by wrapping it into a plain python class. If the model requires a defined input size, an optional function for size preparation can be provided. The pre-defined transformation functions use existing functions from fastai and torchio. misas can be easily extended with custom transformation functions, which require input and output as instances of the Image/ImageSegment fastai classes but can modify the data with arbitrary operations in between.

### Case Study I: Model suitability

The first case study addressed the problem of producing initial training data for a deep learning-based cardiac cine segmentation framework with transfer learning to 7 T [32]. On the one hand there is a public dataset of cardiac magnetic resonance images, the Data Science Bowl Cardiac Challenge (DSBCC) data [33]. But the ground truth labels only contain end-systolic and end-diastolic left-ventricular volumes and not individual segmentation masks. On the other hand, there is a published neural network for cardiac segmentation (further called ukbb_cardiac) [34] which is specifically trained for use with quite homogeneous data from the UK Biobank [35]. Based on this scenario misas was applied to determine the optimal preparation of the DSBCC data to be used by ukbb_cardiac network. [33].

### Case Study II: Model robustness

The second case study showed how sensitivity analysis helps deep learning-based software users to evaluate a newly trained model. More precisely, a model was demanded for segmentation of the heart in transversal ultra-high field MR images to improve B_0_ shimming performance [36]. A model pre-trained on short-axis cine images at 7 T [32] was fine-tuned with very little additional data (90 images from 4 subjects). It was investigated how quickly the segmentation performance collapses when dataset characteristics differ to those of the training set. Furthermore, it was examined which image features are used by the model to make its predictions and what kinds of intuitive or knowledge-based features are learned.

## Results and Discussion

To the best of our knowledge misas is the first tool of its kind. Therefore, there is no systematic comparison and benchmarking with related tools. The following two case studies are presented in detail in the online documentation, including source code, images and graphs (https://chfc-cmi.github.io/misas/). As documentation is written as executable notebooks they can even be interactively explored, without installation using Google Colab. Sensitivity analysis through misas can be performed qualitatively by creating figures with transformed images and overlayed segmentation masks. This can include series of images with different transformation parameters (e.g. rotation angle) or animated gifs. Additionally, quantitative evaluations are possible when ground truth is provided. In this case different scores indicating the quality of segmentation (e.g. Dice) can be calculated along the parameter range and plotted. In the next sections the case studies are only briefly summarized to demonstrate the main points.

### Case Study I: Model suitability

**Fig. 2:**
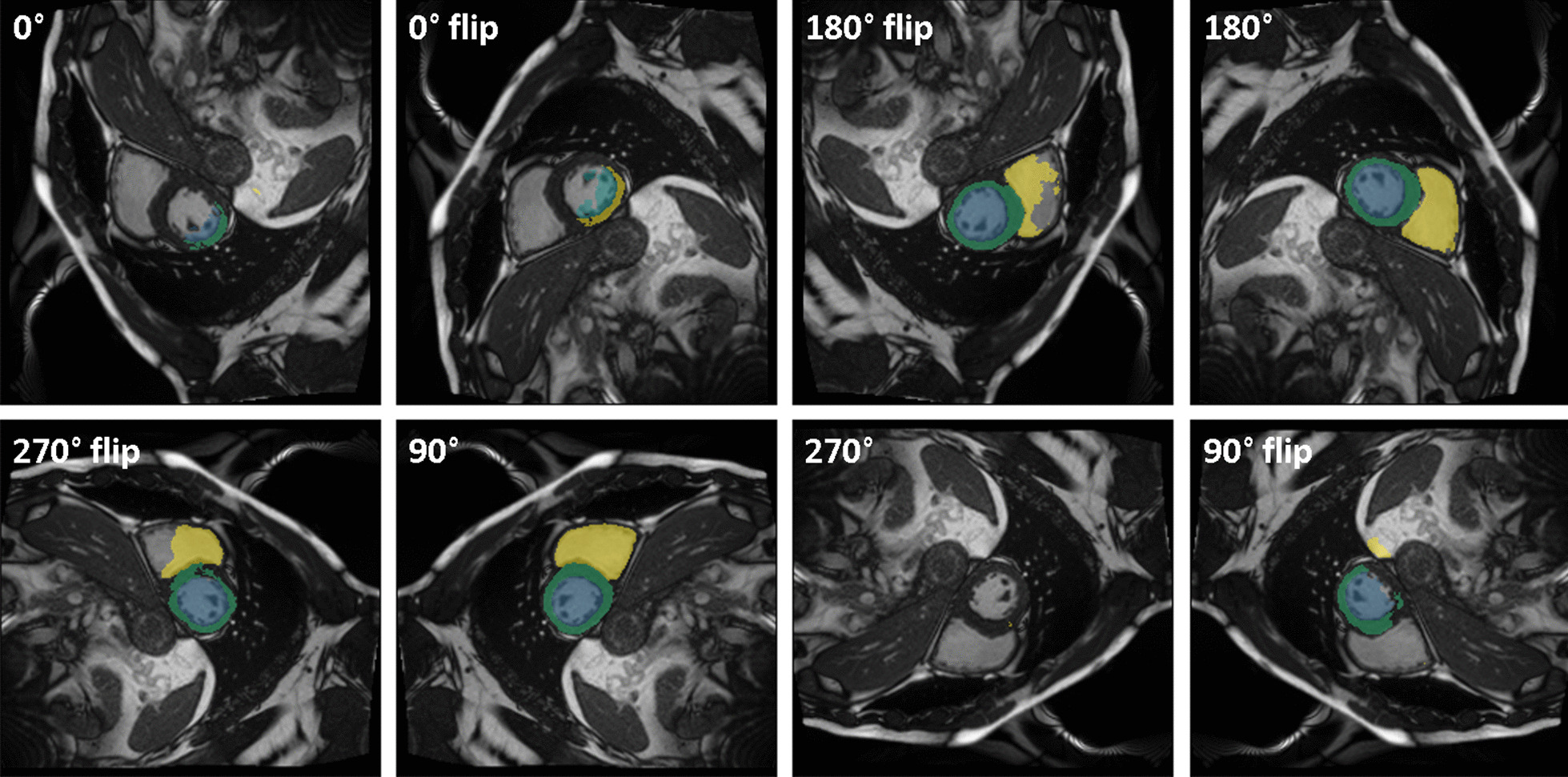
Segmentation result of ukbb_cardiac network [34] on an image from the Data Science Bowl Cardiac Challenge Data [33] on all possible rotations and flips. Performance is highly dependent on image orientation. Rotation angle (clockwise) and flip status (up/down) given

**Fig. 3:**
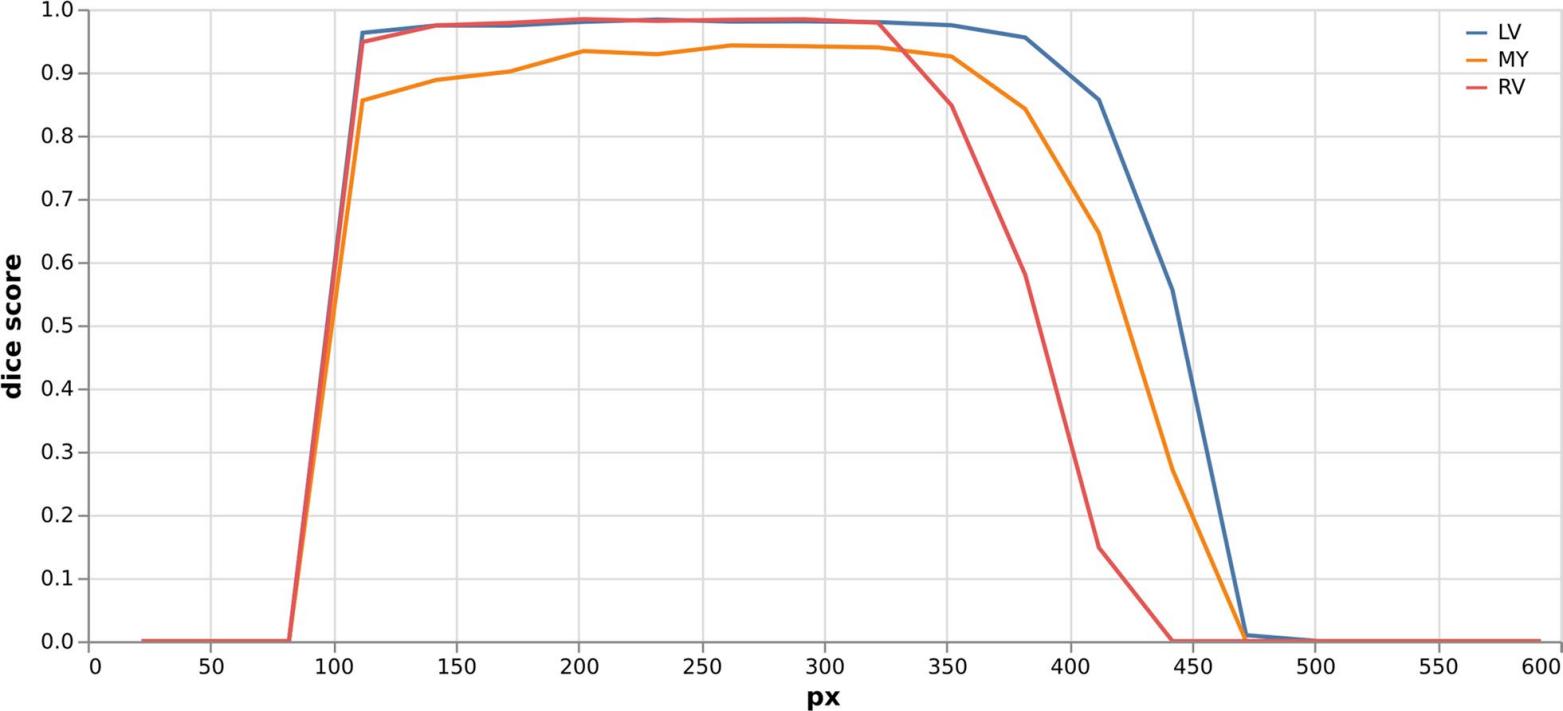
Dice score for each tissue (left ventricle (LV), right ventricle (RV), myocardium (MY)) depending on image size. Small images (< 100px) have 0 dice for all classes, same is true for large images (> 500px). There is quite a broad range ~ 120–320px where predictions are stable

Initial application of the network to random images showed poor performance overall. To improve the performance the impact of image orientation was deciphered in a first step, showing that a rotation by 90° clockwise provided optimal results (Fig. 2). This is equivalent to transposing the axes and flipping left–right and can be explained by the fact that the ukbb_cardiac model usually takes input data from NIfTI format, where axes are stored differently compared to DICOM format. Next the sensitivity to image size becomes apparent as performance breaks down when using images larger than 256 pixels (Fig. 3) or smaller than 100 pixels in either height or width. In between the Dice score of all tissues remains stable on a high level. Further qualitative and quantitative analyses show relatively low sensitivity to other kinds of transformations including brightness, contrast and cropping.

As a result, a clear set of rules for data preparation to optimize prediction accuracy and performance was derived: ideally the images are rotated by 90° and scaled down to 256 pixels.

### Case Study II: Model robustness

**Fig. 4:**
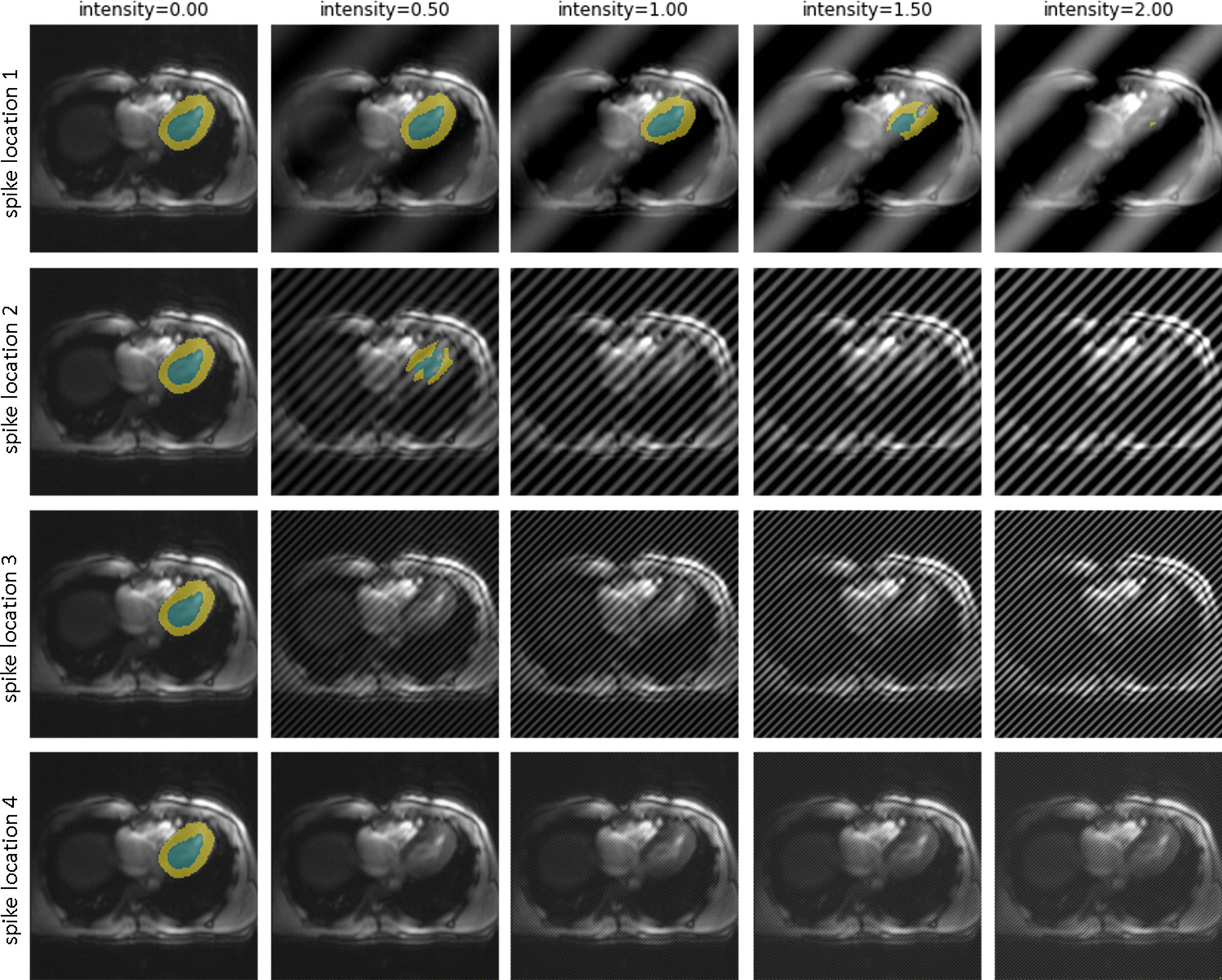
Segmentation performance on transversal slices with simulated spike artifacts of different localization in k-space (rows) and intensity (columns). Intensity parameter denotes the intensity of the spike relative to the original maximum intensity. From top to bottom the location in k-space moves further from the center

An interesting insight, revealed by analysis of sensitivity to rotation is that the model tends to predict the heart on the right-hand side of the image, even incorrectly so when it is rotated by 180°. Additionally, the impact of realistic MR artifacts on sensitivity was analyzed. The analysis of spike artifacts in different positions in k-space and different intensity reveals a high sensitivity (Fig. 4). Only spikes very close to the center of k-space and low intensity are tolerated, all other configurations lead to failure of segmentation.

Overall, the model is quite sensitive to most transformations with only a small parameter range with stable predictions. Quantitatively, this is visible in the Dice score plots as sharp peaks around the neutral parameter value of the transformation. Hence a decision on further training can now be made depending on the use case. As long as the model is used on data locally acquired with identical protocol and no artifacts, the model can be used as is. More data augmentation should be incorporated in re-training for the use on external data. In any case more data is required to further improve segmentation performance.

## General Discussion and Limitations

A major advantage of the developed workflow is its applicability to any model. Access to original training data or anything happening within the blackbox is not required. The only requirement is access to the prediction function. Results of the sensitivity analysis are visualized as overlays on the image or as graphs of a metric over the parameter space. Both visualizations are readily interpretable and easy to understand. Analysis can help to guide decisions like pre-processing of data before usage with a model, or re-training the model with either more or less extensive data augmentation.

While the local interpretability of a single image could easily be analyzed in detail, the obtained information cannot always be transferred to any input image and is a limitation of the presented sensitivity analysis. An image which could be evaluated well should ideally be chosen as the starting point, otherwise unsatisfactory analysis results would be obtained. It might also not be straightforward to derive concrete steps on how the robustness can be improved—or how a specific failure can be eliminated. Moreover, the developed software will not help to evaluate the impact of subtle differences introduced by bias that goes beyond simple transformations (like racial or gender differences). However, if there is a model for artificially introducing a certain kind of bias into an image, the impact of this bias could consequently be analyzed using misas.

It is important to note that sensitivity to a certain transformation is neither a bad nor a good thing per se and must be interpreted in the context of the question at hand.

Furthermore, there is a close relationship between sensitivity analysis and data augmentation. A direct effect between amount and types of data augmentation and model sensitivity regarding the respective transformations is expected. However, sensitivity analysis is still useful for models for which the training process could not be influenced—or even no information on how it was trained could be assessed. Even for self-trained models with data augmentation, sensitivity analysis can be used to check if a suitable amount of data augmentations was employed to reach the desired model robustness.

### Broader applicability and future developments

In the case studies sensitivity analysis was only performed on cardiac MR images. However, neither the method nor the library is restricted to this narrow application area. Both can be applied to other medical imaging areas e.g., cardiac pathology segmentation [37], pneumothorax segmentation or general imaging e.g., CamVid [38] without the need for further adaptions.

Future work will focus on enabling global interpretability by implementing a batch mode that works on multiple example images at once. Additionally, the development of quantitative measures of sensitivity has high priority.

## Conclusions

In this study, we demonstrate how sensitivity analysis can be used to get insights into generic segmentation model performance. It makes predictions more interpretable by expanding the context from single images to a whole range of related images with known transformations. Additionally, we present an open-source python library that allows the scientific community to apply this approach to their own data and models.

### Availability and requirements

- **Project name:** misas.
- **Project home page:** https://github.com/chfc-cmi/misas.
- **Operating system(s):** Platform-independent.
- **Programming language:** Python.
- **Other requirements:** matplotlib, pytorch, fastai (v1.0.61), gif, tensorflow, altair, fastai2, pydicom, kornia, scikit-image, torchio.
- **License:** MIT.
- **Any restrictions to use by non-academics:** None.

## Data Availability

The source code is available in GitHub and zenodo https://github.com/chfc-cmi/misas, https://doi.org/10.5281/zenodo.4106472.

## Abbreviations

API: Application programming interface
DSBCC: Data science bowl cardiac challenge
LV: Left ventricle
Misas: Model interpretation through sensitivity analysis for segmentation
MY: Myocardium
RV: Right ventricle
